# Diagnosis of pulmonary S*cedosporium apiospermum* infection from bronchoalveolar lavage fluid by metagenomic next-generation sequencing in an immunocompetent female patient with normal lung structure: a case report and literature review

**DOI:** 10.1186/s12879-024-09140-3

**Published:** 2024-03-13

**Authors:** Jingru Han, Lifang Liang, Qingshu Li, Ruihang Deng, Chenyang Liu, Xuekai Wu, Yuxin Zhang, Ruowen Zhang, Haiyun Dai

**Affiliations:** 1 Department of Oncology, The First Affiliated Hospital of Chongqing Medical University, No.1 Youyi Road, Yuan Jiagang, Yuzhong District, Chongqing, 400010 China; 2 The First College of Clinical Medicine, Chongqing Medical University, Chongqing, 400016 China; 3 Department of Pathology, School of Basic Medicine, Chongqing Medical University, No.1 Medical College Road, Yuzhong District, Chongqing, 400016 China; 4 The Second College of Clinical Medicine, Chongqing Medical University, Chongqing, 400016 China; 5 Department of Respiratory and Critical Care Medicine, The First Affiliated Hospital of Chongqing Medical University, No.1 Youyi Road, Yuan Jiagang, Yuzhong District, Chongqing, 400010 China

**Keywords:** Metagenomic next-generation sequencing, Pulmonary infection, *Scedosporium Apiospermum*

## Abstract

**Background:**

*Scedosporium apiospermum* (*S*. *apiospermum*) belongs to the asexual form of *Pseudallescheria boydii* and is widely distributed in various environments. *S*. *apiospermum* is the most common cause of pulmonary infection; however, invasive diseases are usually limited to patients with immunodeficiency.

**Case presentation:**

A 54-year-old Chinese non-smoker female patient with normal lung structure and function was diagnosed with pulmonary *S*. *apiospermum* infection by metagenomic next-generation sequencing (mNGS) of bronchoalveolar lavage fluid (BALF). The patient was admitted to the hospital after experiencing intermittent right chest pain for 8 months. Chest computed tomography revealed a thick-walled cavity in the upper lobe of the right lung with mild soft tissue enhancement. *S*. *apiospermum* was detected by the mNGS of BALF, and DNA sequencing reads were 426. Following treatment with voriconazole (300 mg q12h d1; 200 mg q12h d2-d20), there was no improvement in chest imaging, and a thoracoscopic right upper lobectomy was performed. Postoperative pathological results observed silver staining and PAS-positive oval spores in the alveolar septum, bronchiolar wall, and alveolar cavity, and fungal infection was considered. The patient’s symptoms improved; the patient continued voriconazole for 2 months after surgery. No signs of radiological progression or recurrence were observed at the 10-month postoperative follow-up.

**Conclusion:**

This case report indicates that *S*. *apiospermum* infection can occur in immunocompetent individuals and that the mNGS of BALF can assist in its diagnosis and treatment. Additionally, the combined therapy of antifungal drugs and surgery exhibits a potent effect on the disease.

## Background

*Scedosporium apiospermum* (*S*. *apiospermum*) belongs to the asexual form of *Pseudallescheria boydii*, which is widely distributed in various environments. *S*. *apiospermum* is one of the most common causes of invasive fungal infection in patients with immune deficiencies, particularly after organ transplantation, acquired immune deficiency syndrome (AIDS), cystic fibrosis lung disease, structural lung diseases, and long-term use of immunosuppressants or glucocorticoids. The most common infection site of *S*. *apiospermum* is the lungs, and the clinical symptoms are usually cough, expectoration, hemoptysis, fever, dyspnea, and pleuritic chest pain. Imaging changes associated with pulmonary *S*. *apiospermum* infection can be similar to those observed in pulmonary aspergillosis, such as typical fungal balls or non-specific, such as single or multiple nodular lesions with or without cavities, focal infiltration, phyllode infiltration, and bilateral diffuse infiltration. The key to effective treatment is an accurate and timely etiological diagnosis. Otherwise, delayed diagnosis may cause fatal consequences, especially for patients with suppressed immunity. However, it is noteworthy that *S*. *apiospermum* can also rarely infect people with normal immune function, similar to our case. Surgical resection has become an essential part of treatment [1–3]. We presented a rare case of a 54-year-old non-immunocompromised female patient who developed pulmonary *S*. *apiospermum* infection and was diagnosed with pulmonary *S*. *apiospermum* infection by metagenomic next-generation sequencing (mNGS) of bronchoalveolar lavage fluid (BALF), as well as the first literature review of pulmonary *S*. *apiospermum* infection in immunocompetent patients.

## Case presentation

**Fig. 1:**
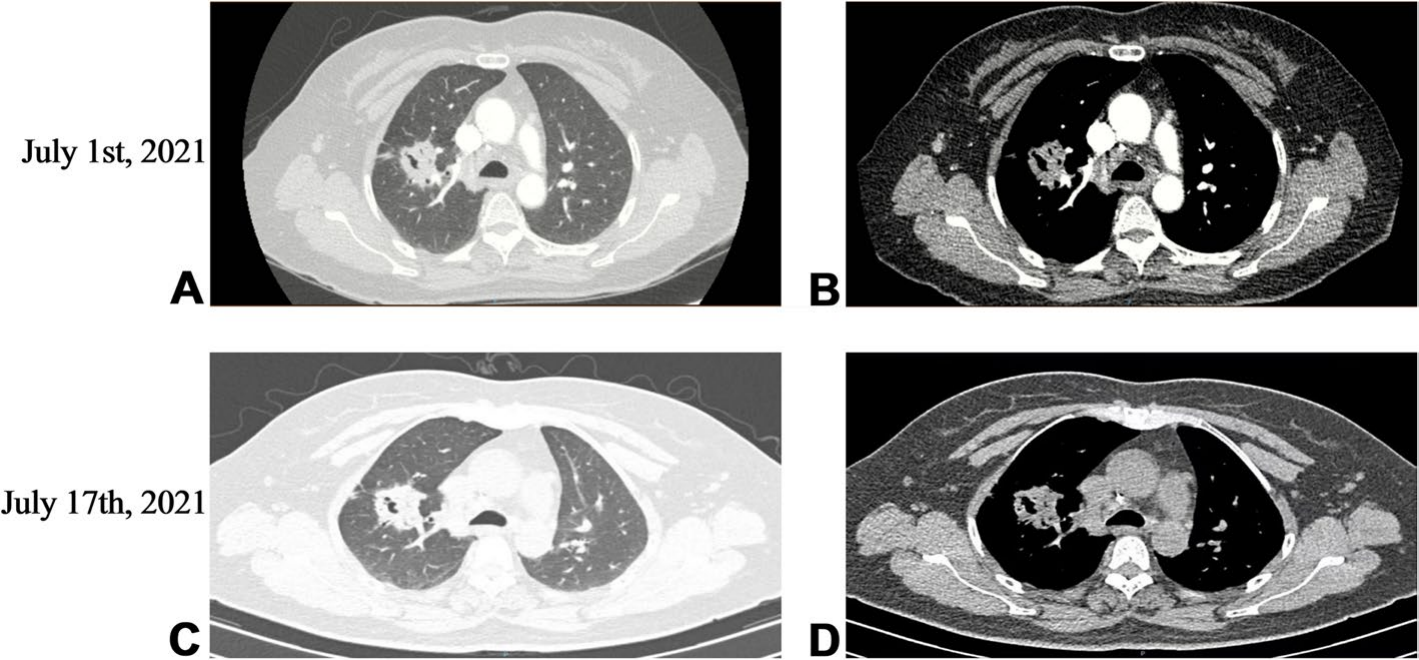
Chest CT at different time points. July 1st, 2021 Chest enhanced CT showed irregular soft-tissue density mass in the upper lobe of the right lung, and the bronchial branch of the upper lobe of the right lung was invaded and narrowed, which suggested a high possibility of lung cancer. There were also several small punctate calcification foci in the left lung (**A**). Slight calcification in mediastinum and left hilar lymph nodes (**B**). July 17th, 2021 After antifungal therapy, CT showed irregular soft tissue density shadow in the upper lobe of the right lung with cavity formation, which was considered to be lung cancer, with little change from the CT result before treatment (**C**, **D**)

**Fig. 2:**
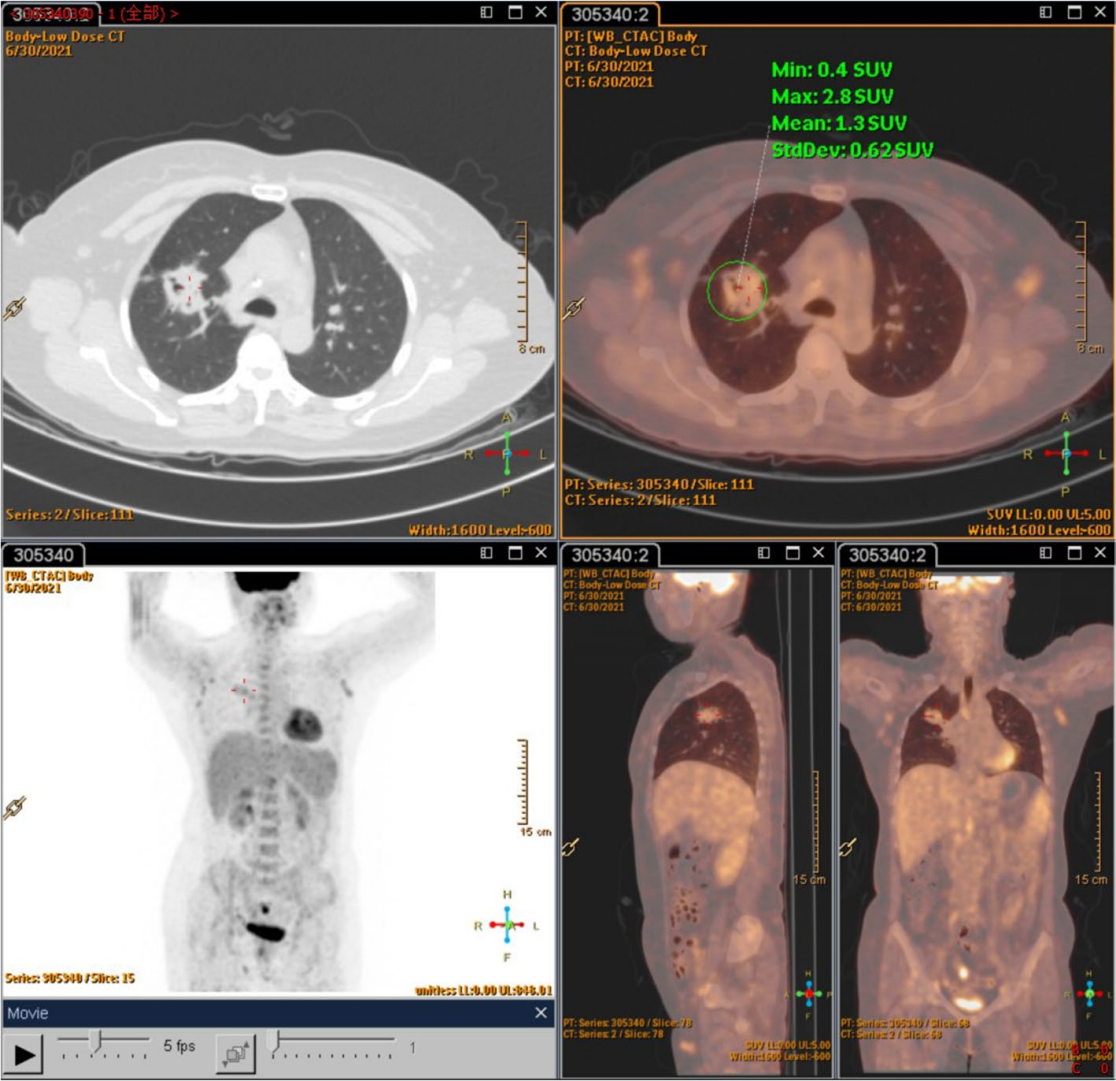
PET-CT. June 30th, 2021 PET-CT indicated space-occupying lesions in the upper lobe of the right lung with increased metabolic activity. Peripheral lung cancer was considered

**Fig. 3:**
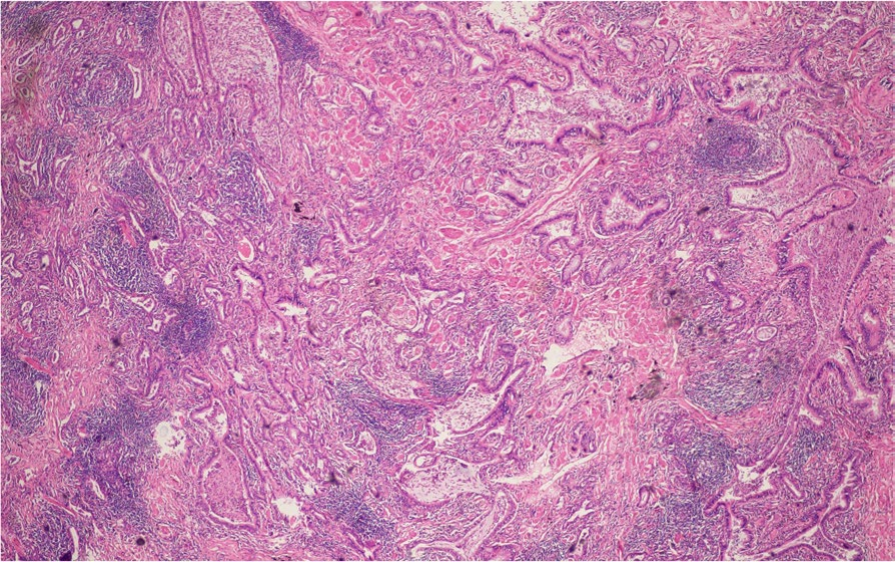
The pathological report of CT-guided percutaneous lung biopsy suggested fibroproliferation with chronic inflammatory cell infiltration

**Fig. 4:**
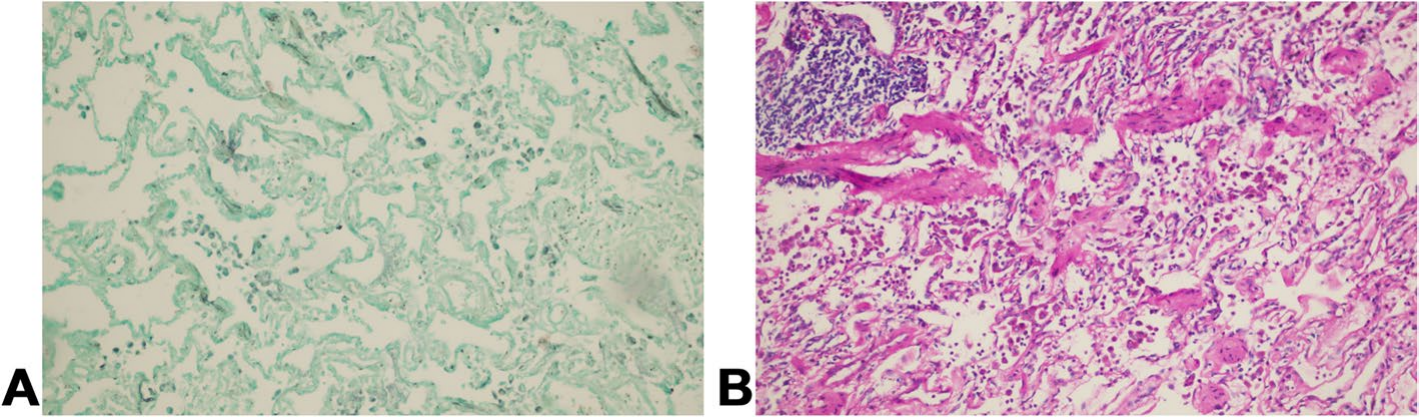
The postoperative pathological results of thoracoscopic right upper lobectomy and lymph node dissection showed that silver staining (**A**) and PAS positive (**B**) oval spores were found in alveolar septum, bronchiolar wall and alveolar cavity, suggesting fungal infection

**Fig. 5:**
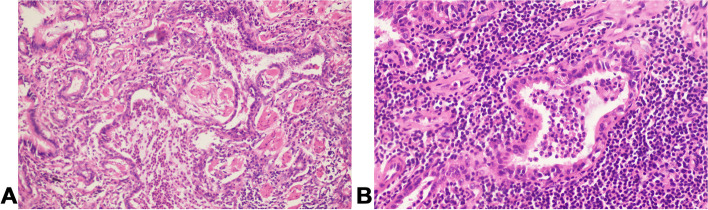
Lung biopsy tissue from the upper lobe of the right lung revealed metaplasia from alveolar to bronchial, along with partial bronchiectasis. In and around the cavity, there was a large amount of inflammatory cell infiltration and foam cell aggregation, accompanied by lymphoid tissue hyperplasia. Fiber hyperplasia was observed in some regions, and alveolar epithelial hyperplasia was also visible

**Fig. 6:**
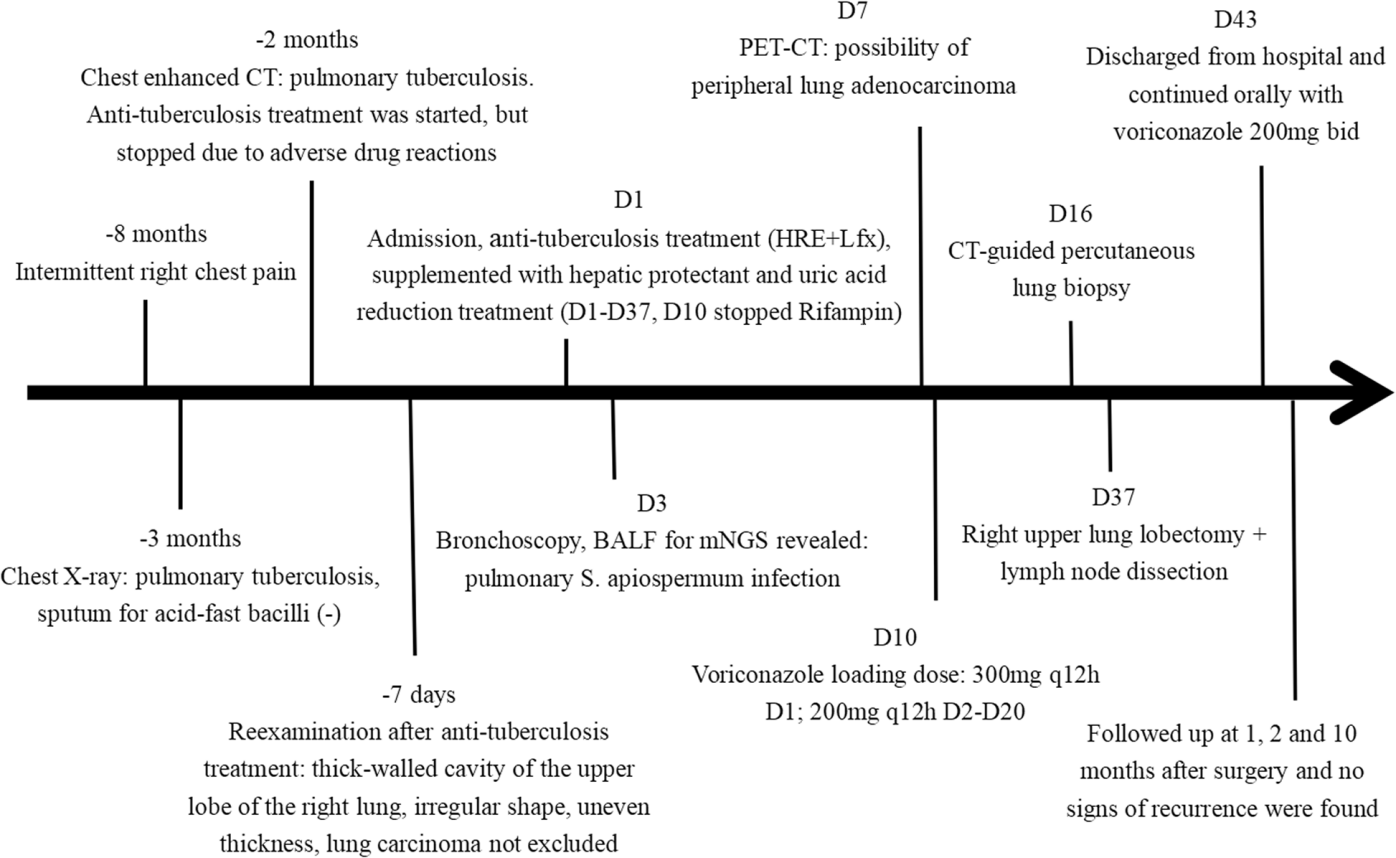
Timeline of events. A flowchart shows the patient’s entire diagnosis and treatment process

**Fig. 7:**
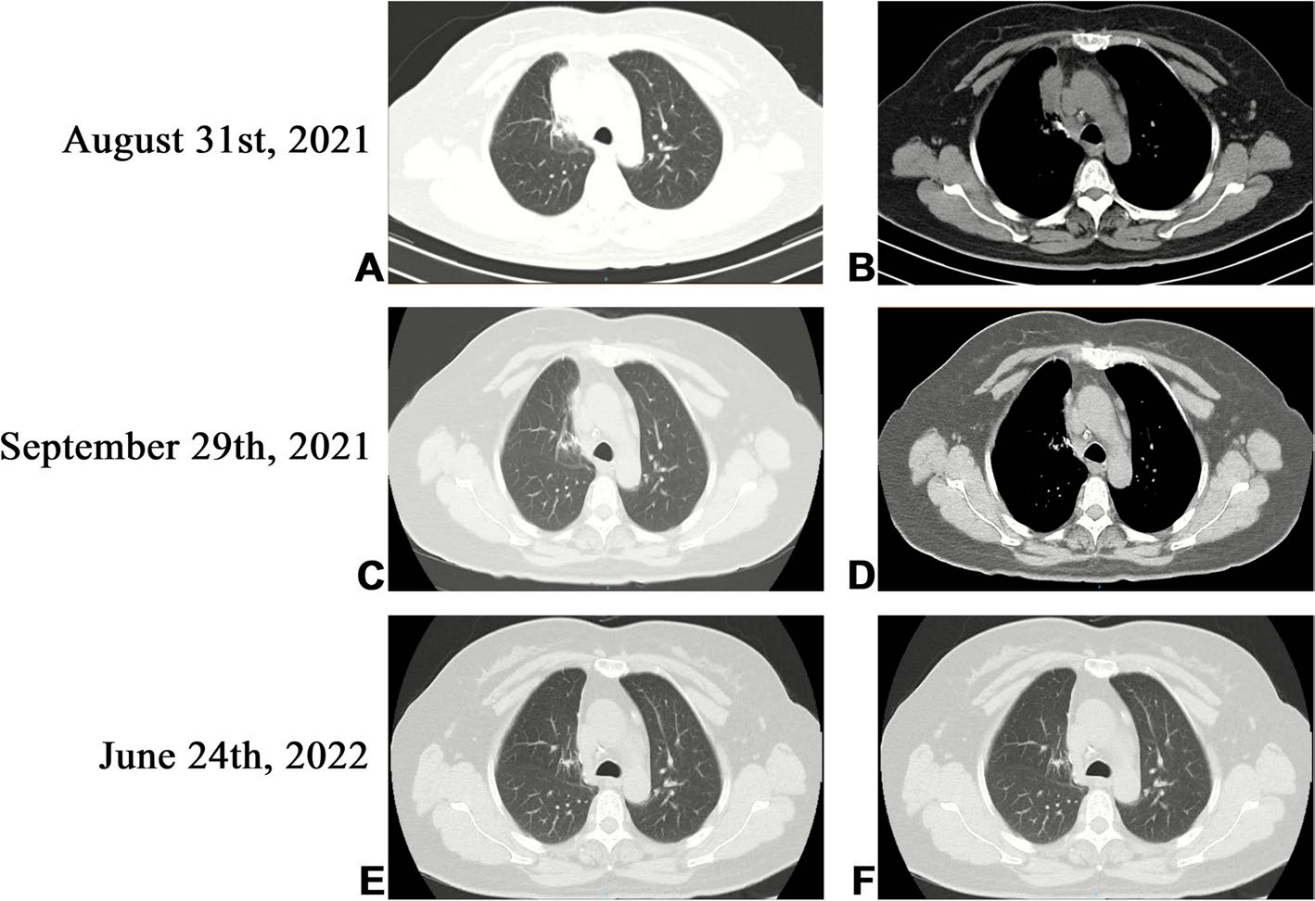
Postoperative reexamination of CT. August 31st, 2021. One month after surgery, HRCT showed the absence of the upper lobe of the right lung, the linear high-density shadow of the right hilar, and the adjacent patchy soft tissue shadow, considering the possibility of postoperative changes. There is a little effusion in the right interlobar fissure (**A**, **B**). September 29th, 2021. Two months after surgery, CT showed the absence of the upper lobe of the right lung, the linear high-density shadow of the right side of the lung, and the adjacent patchy soft tissue shadow, which was slightly smaller than that of 1 month after operation. There was a little effusion in the right interlobar fissure, which was slightly less than before (**C**, **D**). June 24th, 2022. Ten months after surgery, CT showed the absence of the upper lobe of the right lung, the linear high-density shadow in the right hilar area and the adjacent cord shadow, and the soft tissue shadow disappeared 2 months after operation (**E**, **F**)

A 54-year-old Chinese non-smoker female, who worked in a chicken processing factory, experienced intermittent right chest pain with occasional dry cough for 8 months with no apparent trigger. Although the patient’s sputum acid fast staining was negative, she was receiving empirical anti-tuberculosis therapy (2021.04.10) (the specific drug is unknown) in another hospital since her chest computed tomography (CT), dated April 2, 2021, suggested pulmonary tuberculosis. Further, the patient developed skin itching and systemic redness, prompting the anti-tuberculosis drugs to be changed (the specific drug is unknown), however, shortness of breath and shivering occurred following 3 days of medication, and the patient was eventually switched to isoniazid, rifampicin, ethambutol, and levofloxacin (HRE + Lfx) for tuberculosis therapy. Unfortunately, her symptoms and imaging manifestation did not improve, and the CT at Hechuan People’s Hospital on June 16, 2021, indicated a thick-walled cavity in the upper lobe of the right lung, with irregular morphology, uneven wall thickness, and mild soft tissue enhancement. On June 22nd, 2021, she was admitted to our department for further evaluation and treatment. During the investigation, the patient denied experiencing symptoms such as dyspnea, chest tightness, hot flashes, night sweats, hemoptysis, chills, or a high fever. No significant abnormality was observed in the physical examination, and auxiliary inspection results are demonstrated in Table 1. In particular, this patient had underwent the fungal culture of BALF and lung biopsy, but the results were all negative. The patient was initially diagnosed with bacteriologically negative pulmonary tuberculosis and continued with anti-tuberculosis therapy with HRE and Lfx. The BALF of the patient was sent to undergo mNGS analysis, and the mNGS result revealed that the patient suffered from *S. apiospermum* infection, and DNA sequencing reads were 426, followed by antifungal therapy with voriconazole (300 mg iv q12h d1; 200 mg q12h iv d2-d20). Chest enhanced CT suggested the possibility of lung cancer (Fig. 1A and B), and positron emission tomography/CT (PET-CT) indicated that peripheral lung adenocarcinoma was not excluded (SUVmax 2.8) (Fig. 2). No significant improvement was observed in her imaging manifestation after the post-treatment review (Fig. 1C and D), and the possibility of fungal infection along with pulmonary neoplasms was not completely excluded. Then a CT-guided percutaneous lung biopsy was performed, whose pathological report suggested fibroproliferation with chronic inflammatory cell infiltration (Fig. 3). Although no evidence of pulmonary neoplasms was observed during the lung biopsy, a thoracoscopic right upper lobectomy and lymph node dissection were performed. During the surgery, no pleural effusion was observed, and the lesion was located in the upper lobe of the right lung, measuring about 3*3 cm, with complete excision of the diseased lobe. The postoperative pathological results revealed visible silver-stained (Fig. 4A) and PAS-positive (Fig. 4B) oval spores in the alveolar septum, bronchiole wall, and alveolar cavity, thus, indicating fungal infection. Lung biopsy tissue from the upper lobe of the right lung revealed metaplasia from alveolar to bronchial, along with partial bronchiectasis. In and around the cavity, there was a large amount of inflammatory cell infiltration and foam cell aggregation, accompanied by lymphoid tissue hyperplasia. Fiber hyperplasia was observed in some regions, and alveolar epithelial hyperplasia was also visible (Fig. 5). The patient continued to consume voriconazole (200 mg po bid) for 2 months after surgery, and the diagnosis and treatment process are indicated in Fig. 6. Chest imaging was followed up at 1, 2, and 10 months after surgery, and no signs of recurrence were observed (Fig. 7).

**Table 1:** Detailed auxiliary inspection results

Parameter	Result/Value
CBC	normal
Hepatic function	normal
Coagulation function	normal
Scr	normal
BUN	normal
Stool routine	normal
Urine routine	normal
**Tuberculosis related**	
PPD	negative
TB-Ab	negative
T-SPOT	negative
X-PERT	negative
Acid-fast bacilli in sputum	negative
Non-tuberculous mycobacteria	negative
**Infection related**	
CRP	normal
PCT	normal
ESR	normal
Nine respiratory pathogens	negative
**Tumor related**	
CYFRA21-1	3.4ng/ml(0-3.3)
ProGRP	42.6pg/ml(25.3–77.8)
SCC	1.2ng/ml(0-2.7)
CEA	1.7ng/ml(0.2–10.0)
NSE	12.1pg/ml(0-16.3)
**Fungi related**	
GM(plasma) test	negative
G(plasma) test	negative
GM(BALF) test	1.24
BALF culture	No fungal growth detected after 7-day culture
Smear of lung biopsy	No fungi found
**Immune status**	
HIV	negative

## Literature review

**Fig. 8:**
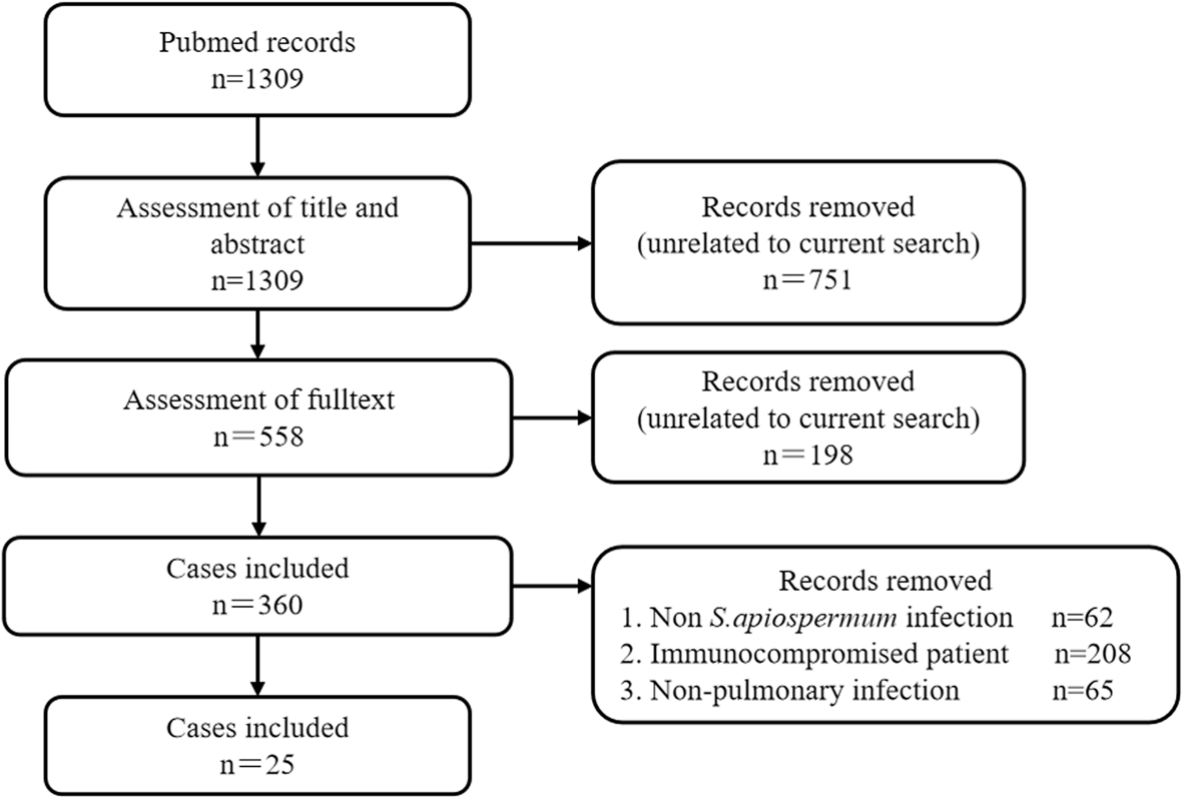
Screening process. The flow chart shows the process of literature review

We searched the keywords “Pulmonary” or “Lung” and “Scedosporium” or “Scedosporiums” or “Scedosporium apiospermum” on the PubMed database, which had a total of 1309 articles. Subsequently, we excluded studies unrelated to current research, patients without *S. apiospermum* infections, patients with immunocompromised lung, and patients with non-pulmonary infections. Finally, 25 medical records with complete case report data were retrospectively analyzed [4–27]. The flow chart of the screening process is indicated in Fig. 8. The included patients were elaboratively summarized per the age, sex, major clinical manifestations, presence or absence of pre-existing disease, diagnostic methods, imaging manifestations, extensive or limited lesions, presence or absence of delay in diagnosis and treatment, treatment plan, and treatment outcome (Table 2).

**Table 2:** Brief summarization of included patients

Characteristics	25 patients
Sex (male / female)	13/12
Median age (year)	55 (7–83)
Underlying disease	21 (0.84)
Pulmonary tuberculosis	11 (0.44)
Pulmonary cystic fibrosis	4 (0.16)
Bronchiectasis	3 (0.12)
Previous diagnosis of *S. apiospermum*	1 (0.04)
Pulmonary arterial hypertension	1 (0.04)
Diabetes	1 (0.04)
Previous tumor history	1 (0.04)
Oral abscess	1 (0.04)
No underlying disease	4 (0.16)
Clinical manifestations	
Cough, Expectoration	16 (0.64)
Hemoptysis	12 (0.48)
Fever	10 (0.40)
Dyspnea	9 (0.36)
Night sweats	3 (0.12)
Blood in sputum	2 (0.08)
Weight loss	3 (0.12)
Loss of appetite	2 (0.08)
Chest pain	2 (0.08)
Fatigue	2 (0.08)
Pneumothorax	1 (0.04)
Diagnosis time	
Misdiagnosed	9 (0.36)
No misdiagnosis	16 (0.64)
Diagnostic methods	
BALF culture	13 (0.52)
Sputum culture	7 (0.28)
Blood culture	1 (0.04)
Lung biopsy tissue smear	1 (0.04)
Lung biopsy tissue specimen culture	2 (0.08)
Postoperative tissue culture	4 (0.16)
DNA/RNA sequencing of BALF	2 (0.08)
Lesion range	
Limited	12 (0.48)
Extensive	13 (0.52)
Treatment scheme	
Antifungal therapy	15 (0.60)
Surgery treatment	4 (0.16)
Antifungal + Surgery	6 (0.24)
Prognosis	
Cure	19 (0.76)
Improvement	2 (0.08)
Death	2 (0.08)
No mention	2 (0.08)

A total of 25 immunocompetent patients with pulmonary *S*. *apiospermum* infection were reported on PubMed, and the basic characteristics of these patients are summarized in Table 3. In total, 12 females (48%) and 13 males (52%) were included; the average age of the patients was 50.96 years, ranging from 7 to 83 years. Of the included patients, 84% had the following symptoms: 16 with cough and expectoration (64%), 12 with hemoptysis (48%), 10 with fever (40%), nine with dyspnea (36%), and other symptoms including night sweats (12%), weight loss (12%), chest pain (8%), blood in the sputum (8%), anorexia (8%), fatigue (8%), and pneumothorax (4%). Cough and expectoration were the most common symptoms, followed by hemoptysis and fever. The severity of symptoms also varied, from inconspicuous pulmonary symptoms (three cases) to dyspnea (nine cases). Pulmonary tuberculosis was the most common underlying disease (11/25, 44%), followed by pulmonary cystic fibrosis (4/25, 16%) and bronchiectasis (3/25, 12%). The main diagnostic method was BALF culture in 13 cases (52%), followed by sputum culture in seven cases (28%), postoperative tissue culture in four cases (16%), lung biopsy and transbronchial lung biopsy in two cases (8%), gene sequencing of alveolar lavage fluid in two cases (8%), blood culture in one case (4%), and lung biopsy smear in one case (4%). Among the 25 patients included, four (16%) were treated with surgery, 15 (60%) with antifungal therapy (including one case with combined nebulized dornase Alfa and 7% hypertonic saline), and six (24%) were treated with surgery combined with antifungal therapy. Among these patients, treatment was delayed for nine (36%) patients, of which four (16%) were misdiagnosed as *Aspergillus* infections, one (8%) had empirical tuberculosis treatment, one (8%) whose prior bronchoalveolar lavage culture had later grown *S. apiospermum* and had been considered a contaminant, one patient (8%) had an unidentified fungus isolated from lung puncture biopsy, one patient (8%) had *S. apiospermum* detected in sputum three years prior deterioration, but the finding was disregarded, and one case (8%) was treated with antibiotics without finding etiological evidence. Fortunately, all of the above nine patients with delayed diagnosis were effectively treated after diagnosis of *S. apiospermum* infection. Out of the 25 reported cases, prognosis was not mentioned in two cases, in the remaining 23 cases mortality rate was 8.7% (2/23), cure rate was 82.6% (19/23), and 8.7% (2/23) of the patients showed improvement in their conditions. Of the cases that received only antifungal therapy, one died (6.7%, 1/15 cases). All four patients who received only surgical therapy were cured. Of the patients who received surgery in combination with antifungal therapy, treatment was effective in five cases (83.3%, 5/6 cases). The majority of hosts with normal immune function had a favorable prognosis, however, factors such as prolonged disease duration, underlying diseases, and delayed diagnosis and treatment may have caused the death.

**Table 3:** Basic characteristics of included patients

Case load	Age /Gender	Symptom	Past medical history and associated risk factors	Diagnosis	Imaging performance	Limit/extensive	Delay diagnosis or not	Therapy	Outcome	The year of publication
1 [4]	44/female	Hemoptysis Cough Blood in sputum Weight loss Anorexia	None	BALF culture	Hollow lesion in the left upper lobe, Bronchiectasia	Limit	Yes, Anti-TB Antibiotic treatment	Voriconazole → Surgery→ Voriconazole	Cure	2020
2 [5]	73/female	None	None	BALF and TBLB sample culture	Single bossing	Limit	No	Surgery	Cure	2018
3 [6]	72/male	Fever Hemoptysis	TB at the age of 30 years	Sputum culture	Hollow lesion pulmonary infiltration, Air crescent sign	Extensive	Yes, Misdiagnosed as aspergillus infection	Miconazole	Not mention	2005
4 [7]	24 /male	Chronic cough Expectoration Intermittent Hemoptysis	Tooth decay recurrent oral abscesses	Lung biopsy culture	Hollow lesion typical of a fungal ball	Limit	Yes, Antibiotic treatment	Itraconazole →Surgery	Not mention	2005
5 [8]	47 /male	Hemoptysis Cough Expectoration Dyspnea	TB for 6 years	Sputum culture	Fungal bulb, Bilateral uneven infiltrating foci	Limit	No	Itraconazole →Surgery	Cure	2014
6 [9]	59 /female	Fever	None	BALF culture and DNA sequence	Infiltrates and nodular lesions on both sides of the lungs	Extensive	Yes, Misdiagnosed as aspergillus infection, treated with micafengin, in parallel with empiric antimicrobial therapy	Voriconazole, Liposomal Amphotericin	Improve	2011
7 [10]	26 /male	Cough Expectoration Fever Spontaneous Pneumothora Fungal empyema	*S. apiospermum* infection	BALF culture	Bronchiectasia Multiple cavities with nodules Enlarged mediastinal lymph nodes	Extensive	No	Posaconazole →Surgery→ Posaconazole	Cure	2011
8 [11]	40/male	Cough Hemoptysis	TB for 15 years	Postoperative specimens culture	Typical fungal balls Air crescent sign	Extensive	Yes, Misdiagnosed as TB and Aspergillus infection	Voriconazole	Cure	2016
9 [12]	51/female	Dry cough Night sweats	None	BALF culture	Hollow lesion Airway dilation	Limit	Yes, considered as contaminant	Voriconazole → Surgery	Cure	2017
10 [13]	83/female	Cough Blood in sputum Fatigue Dyspnea	Bronchiectasia COPD Chronic atrial fibrillation	BALF culture	Bronchiectasia Tree bud sign	Limit	No	Voriconazole	Cure	2021
11 [14]	72/female	Hemoptysis Fever Polypnea	Pulmonary arterial hypertension	Boold culture	Both lungs are scattered in blurred patches	Extensive	No	Voriconazole and Amphotericin B →Terbinafine	Cure	2020
12 [15]	67/male	Hemoptysis Fever	Non-tuberculous Mycobacte for 15 years	BALF culture	Fungal sphere cavular lesions	Limit	No	Voriconazole	Cure	2021
13 [16]	67/male	Cough Hemoptysis Dyspnea	Bronchiectasia TB	BALF culture	Hollow lesions Bronchiectasia Tree bud sign	Extensive	Yes, Antibacterial therapy	Itraconazole Voriconazole	Cure	2015
14 [17]	71/male	Fever Cough Expectoration	TB Hypertension	BALF culture and lung tissue biopsy smear	Hollow lesions Fungal sphere-like shadows	limit	Yes, Misdiagnosed as Aspergillus	Voriconazole	Cure	2011
15 [18]	74/female	None	Mycobacterium tuberculosis avium infection	BALF culture	Bronchiectasia, Cavity, Nodules	Extensive	No	Voriconazole	Cure	2020
16 [19]	54/female	Fever Dry cough Dyspnoea Weight loss	TB	Sputum culture	Left lower lung infiltration and diffuse small nodular infiltration in the right lung	Extensive	No	Miconazole nitrate Ketoconazole	Cure	1997
17 [20]	68/male	Cough Purulent sputum Hemoptysis Night sweats Fever Dyspnea Weight loss Fatigue Anorexia	TB 40 years before	Sputum culture	A thick walled cavity with necrosis	Extensive	No	Voriconazole →Surgery	Death	2007
18 [21]	36/female	Chest pain Fever Cough Purulent sputum Dyspnea	DM	Postoperative tissue culture	A nodular mass with meniscus sign in the right lower lobe with undefined border	Limit	No	Surgery	Cure	2004
19 [22]	57/female	Right-side chest pain Hemoptysis	TB	Postoperative tissue culture	Partial fibroatelectasic retraction of the left upper lobe and a thin-walled cavity	Limit	No	Surgery	Cure	2004
20 [22]	61/female	Cough Hemoptysis	TB	Sputum culture and BLAF culture	Numerous cavities with indwelling fungal balls Bronchiectasis	Extensive	No	Voriconazole and Bronchial artery embolism	Cure	2011
21 [23]	55/male	none	History of bladder cancer	Post-operative tissue culture	Air crescent sign	Limit	No	Surgery	Cure	2002
22 [24]	17/male	Hemoptysis Respiratory failure	Pulmonary cystic fibrosis	BALF Gene sequencing	Severe spongous lung destruction	Extensive	Yes, Delayed treatment	Venous Voriconazole and Liposomal Amphotericin B→nebulidized Voriconazole and intravenous Voriconazole	Cure	2014
23 [25]	37/male	Cough Expectoration	Pulmonary cystic fibrosis Bronchiectasia	Sputum culture	Bronchiectasis Air crescent sign Hollow lesions	Extensive	No	Intravenous Voriconazole → nebulidized Voriconazole and Amphotericin B	Death	2010
24 [26]	7/female	Fever Dyspnea Cough	Pulmonary cystic fibrosis	BALF culture and sputum culture	Multiple bronchiectasis and bronchial thickening	Extensive	No	Amphotericin B Itraconazole	Cure	2006
25 [27]	12/male	Dry cough Dyspnea	Pulmonary cystic fibrosis	BALF culture	Peribronchial thickening in lower lobes	Limit	No	Voriconazole → nebulidized dornase Alfa and 7% hypertonic saline	Improve	2015

## Discussion and conclusions

*S*. *apiospermum* is widely distributed in various environments, such as contaminated water, wetlands, sewage, and saprobic heritage [28]. Most of the infections occur in patients with immune deficiency, such as those with AIDS, malignant tumors, long-term use of immunosuppressants or glucocorticoids, and organ transplantation, which can cause fatal disseminated infection [29–32]. Additionally, it can occur in patients with normal immune function [4–27]. Our literature review revealed that 11 patients (44%) had a history of pulmonary tuberculosis infection, which was consistent with Kantarcioglu et al.‘s hypothesis that pulmonary tuberculosis infection was the main risk factor for *S*. *apiospermum* pulmonary infection [33].

It has been reported that the risk factors for *S*. *apiospermum* infection in immunocompromised patients include lymphopenia, neutropenia, and serum albumin levels of < 3 mg/dL [30]. In immunocompetent patients, the main risk factors for *S*. *apiospermum* infection are surgery or trauma [34], and the lung and upper respiratory tract are the most infected sites. These infections fall into the following categories: Transient local colonization, bronchopulmonary saprobic involvement, fungus ball formation, and invasive *S*. *apiospermum* pneumonia [1]. Among the clinical features of *S*. *apiospermum* pulmonary infection, fever is the most common clinical sign and symptom in most cases, and other common symptoms are cough, expectoration, hemoptysis, dyspnea, and pleuritic chest pain [35]. The imaging manifestations of *S*. *apiospermum* pulmonary infection are similar to those of other infections, such as the formation of fungus balls in preexisting cavities, which is difficult to differentiate from an *Aspergillus* ball using radiograms. It may also exhibit solitary or multiple nodular lesions with or without cavitation, focal, lobar, or bilateral diffused infiltration [1]. Consistent with our literature review, *S*. *apiospermum* infection is frequently misdiagnosed as pulmonary aspergillosis or tuberculosis given the non-specific imaging features [6, 9, 11, 17]. The imaging of our patient presented thick-walled cavities in the right upper lobe, with uneven thickness, an irregular shape, and adjacent pleural adhesion, which are non-specific for pulmonary infections caused by *S. apiospermum*. Additionally, this patient experienced intermittent right chest pain and occasional dry cough for 8 months without an obvious trigger, which is consistent with tuberculosis symptoms. Prior to admission to our hospital, X-ray and CT conducted at another hospital was suggestive of pulmonary tuberculosis. Therefore, since tuberculosis was highly suggested based on symptoms and imaging, with no apparent risk factors for fungal infections, empirical anti-tuberculosis therapy was initiated prior to antifungal therapy consistent with other reports [4, 36, 37].

*S*. *apiospermum* infection can be diagnosed by microbiology (including direct staining and culture), histopathology, and polymerase chain reaction to identify fungal DNA [38–41]. Additionally, serology can aid in the diagnosis as *S. apiospermum* infection through antigen detection using counter-immunoelectrophoresis and enzyme-linked immunosorbent assay [42, 43]. However, owing to the cross-reactions with antigens from other fungi such as *Aspergillus* spp, this method was not reported in cases [43]. To the best of our knowledge, this is the first reported case to use mNGS of BALF in the diagnosis of pulmonary *S*. *apiospermum* infection. Delays in diagnosis and treatment of *S. apiospermum* infection can be fatal, particularly in immunocompromised patients. mNGS can provide rapid and reliable method and offer a valuable diagnostic support, thereby avoiding delays in diagnosis and treatment.

In immunocompromised patients, infections caused by *S*. *apiospermum* are difficult to treat and usually fatal, whereas immunocompetent hosts had a better prognosis [1]. *S*. *apiospermum* infection is difficult to treat as it has been reported to be resistant to many antifungal agents, such as fluconazole, ketoconazole, flucytosine, terbinafine, itraconazole, and liposomal amphotericin B, however, it is susceptible to voriconazole, and a few studies have reported its efficacy in the treatment of *S*. *apiospermum* infection [5, 29, 44, 45]. According to the literature, surgical excision is an effective treatment for infections caused by *S. apiospermum* when lesions are localized [1]. Even in immunocompetent patients, infections caused by this pathogen often require surgical excision [1]. According to Liu et al.‘s a meta-analysis and systematic review of pulmonary *S*. *apiospermum* infection, more than half of the immunocompetent patients with pulmonary infection received surgical treatment, however, this did not cause a better overall survival rate [46]. However, since antifungal therapy failure is more common in immunocompromised patients, surgical resection may help to improve survival rates, whereas immunocompetent patients treated with antifungal therapy alone may have a good prognosis [46]. The overall mortality for pulmonary *S. apiospermum* infection in patients with the normal immune function was 12.5% (5/40), and among them who received surgery, the mortality was 9.09% (2/22), while the patients without surgery had a mortality of 16.67% (3/18) [46]. Our literature review revealed that the total mortality, the rate of patients who were cured, and improvement rates of 25 patients with normal immune function were 8.7%, 82.6%, and 8.7%, respectively. One patient who received antifungal treatment alone died (6.7%, 1/15), whereas four patients who received surgical treatment were cured, and five patients (83.3%, 5/6) responded favorably to surgery combined with antifungal therapy. In this case, the patient’s immune function and lung structure were normal. The reasons for surgical excision were as follows: (1) Based on the PET-CT report, it was suggested that the local metabolic activity of the upper lobe of the right lung was high (SUVmax = 2.8), and peripheral gonadal carcinoma was suspected; (2) Following antifungal treatment, the foci were not healed, and the possibility of pulmonary fungal infection complicated with lung cancer could not be excluded. The surgery aimed to remove the foci and actively resect the pulmonary tumor simultaneously based on a reported case of pulmonary *S*. *apiospermum* infection with pulmonary tumorlets in an immunocompetent patient [5]. The patient was followed up for 10 months after surgery, and the symptoms of dry cough and chest pain had improved, with the chest CT indicating effective treatment.

Based on our case and literature review, despite the absence of trauma or surgery, people with normal immune function and lung structure can also be infected with *S*. *apiospermum*. This case highlights mNGS in the clinical diagnosis of pulmonary invasive fungal disease. For traditional culture fail to provide clear pathogenic evidence, it is a rapid and reliable test to avoid the adverse consequences of delayed diagnosis and treatment. The combination of antifungal therapy and surgery is effective in the treatment of local lesions of pulmonary infection caused by *S*. *apiospermum* in hosts with normal immune function, especially when patients suffers from *S. apiospermum* infection combined with tumors.

## Data Availability

The datasets used and analysed during the current study available from the corresponding author on reasonable request.

## Abbreviations

*S. apiospermum*: *Scedosporium apiospermum*
mNGS: Metagenomic next-generation sequencing
BALF: Bronchoalveolar lavage fluid
AIDS: Acquired immune deficiency syndrome
CT: Computed tomography
PET-CT: Positron emission tomography/computed tomography
